# Transforming Growth Factor Beta Receptor 3 Haplotypes in Sickle Cell Disease Are Associated with Lipid Profile and Clinical Manifestations

**DOI:** 10.1155/2020/3185015

**Published:** 2020-10-13

**Authors:** Rayra P. Santiago, Camylla V. B. Figueiredo, Luciana M. Fiuza, Sètondji C. M. A. Yahouédéhou, Rodrigo M. Oliveira, Milena M. Aleluia, Suellen P. Carvalho, Cleverson A. Fonseca, Valma M. L. Nascimento, Larissa C. Rocha, Caroline C. Guarda, Marilda S. Gonçalves

**Affiliations:** ^1^Laboratório de Investigação em Genética e Hematologia Translacional, Instituto Gonçalo Moniz, FIOCRUZ-BA, Salvador 40296-710, Brazil; ^2^Universidade Federal da Bahia, UFBA, Salvador 40170-110, Brazil; ^3^Departamento de Ciências Biológicas, Universidade Estadual de Santa Cruz, UESC, Ilhéus 45662-900, Brazil; ^4^Fundação de Hematologia e Hemoterapia do Estado da Bahia, HEMOBA, Salvador 40286-240, Brazil

## Abstract

Individuals with sickle cell disease (SCD) present both chronic and acute inflammatory events. The TGF-*β* pathway is known to play a role in immune response, angiogenesis, inflammation, hematopoiesis, vascular inflammation, and cell proliferation. Polymorphisms in the transforming growth factor-beta receptor 3 (*TGFBR3*) gene have been linked to several inflammatory diseases. This study investigated associations between two *TGFBR3* haplotypes and classical laboratory parameters, as well as clinical manifestations, in SCD. We found that individuals with the GG haplotype presented higher levels of total cholesterol (TC), low-density lipoprotein cholesterol (LDL-C), triglycerides, non-HDL cholesterol, total proteins, and globulin than individuals with non-GG haplotypes. In addition, the GG haplotype was associated with a previous history of pneumonia. Individuals with the CGG haplotype presented increased plateletcrit, TC, LDL-C levels, and non-HDL cholesterol. The CCG haplotype was also associated with a previous history of pneumonia. Our findings suggest that individuals with the GG and CGG haplotypes of *TGFBR3* present important alterations in lipid profile.

## 1. Introduction

Sickle cell disease (SCD) is an autosomal genetic disorder marked by a chronic inflammatory status with acute episodes. The acute condition occurs as a result of vaso-occlusion, persistent cycles of red blood cell sickling and unsickling, and hemolysis, leading to leukocyte and endothelial cell activation that induces the release of cytokines and adhesion molecules. By contrast, the chronic inflammatory status is the result of ischemic and reperfusion processes, which cause endothelial cell and vascular wall damage [1–3].

The clinical complications, which occur frequently in individuals with SCD, are classified as acute or chronic according to the age of affected individuals, yet are not restricted to any specific stage of life [4, 5]. SCD individuals also present clinical manifestations including vaso-occlusive (VO) and painful crises, pneumonia, cholelithiasis, stroke, priapism, and chronic injury in a variety of organs [6]. One common acute complication in SCD is acute chest syndrome (ACS), characterized by cough, shortness of breath, and signs of hypoxemia that are difficult to distinguish from acute pneumonia [7]. Pulmonary complications in SCA are mostly associated with vascular impairment and vasoconstriction, leading to VO [8]. Among the clinical manifestations associated with hemolysis, cholelithiasis, which is related to gallstone formation and gallbladder obstruction, tends to increase in frequency with age [9]. Cholelithiasis occurs due to the accelerated rate of chronic erythrocyte destruction in individuals with SCD. Heme is released by hemolysis and becomes metabolized into bilirubin, which can form insoluble calcium bilirubinate in the bile and precipitate as pigments that form gallstones [10].

The immunological aspects of SCD have been widely studied among individuals with sickle cell anemia (SCA), with high levels of cytokines detected, including interleukin (IL) 4, 6, 8, 10, and tumor necrosis factor-alpha (TNF-a) [11]. In addition, transforming growth factor-beta (TGF-*β*) and IL-17 were also found to be associated with vascular activation and inflammation based on a direct association with arginase levels [12]. Differences were found in the levels of cytokines (IL1*β*, IL6, TNF-a, and TGF-*β*), lipid inflammatory mediators (LTB4 and PGE2), and modulators of vascular remodeling (MMP9 and TIMP1) between SCA individuals in steady- and crisis-state, permitting the characterization of these two groups using these parameters [13].

The TGF-*β* pathway is involved in several cellular processes, since signal transduction involves binding with transforming growth factor- (TGF-) beta receptors (TGF*β*RI, TGF*β*RII, or TGF*β*RIII), which activate mothers against decapentaplegic homolog (SMAD) proteins and other mediators. As TGF-*β* is known to activate several mediators, the TGF-*β* pathway is considered to play a role in immune response, angiogenesis, inflammation, hematopoiesis, vascular inflammation, and cell proliferation [12, 14].

The transforming growth factor-beta receptor 3 (*TGFBR3*) gene encodes a receptor of the transforming growth factor-beta (TGF-*β*) family, the TGF-*β* type III receptor (T*β*RIII), which presents affinity with all three TGF-*β* isoforms [15]. Polymorphisms in gene *TGFBR3* have been linked to several diseases, such as Marfan syndrome, bladder cancer, Behçet's disease, and SCD [14, 16–18]. In individuals with SCD, some polymorphisms have been associated with stroke, leg ulcers, priapism, pulmonary hypertension, osteonecrosis, and acute chest syndrome, all severe clinical manifestations [19–25].

Considering the complex mechanisms underlying the pathogenesis of SCD, we sought to investigate associations between *TGFBR3* haplotypes and classical laboratory parameters, as well as clinical manifestations.

## 2. Materials and Methods

### 2.1. Subjects

One hundred seventy-five individuals with SCD (HbSS and HbSC genotypes) were seen at the Bahia Hemotherapy and Hematology Foundation between October 2016 and September 2017. The individuals, 83/175 (47.4%) of whom were female, had an average age of 14.46 ± 3.35 years and a median age of 14 years (interquartile range [IQR]: 12-17 years). All individuals with SCD were in steady-state, characterized by the absence of acute crisis in the three months prior to blood collection procedures. None of the patients were undergoing therapy with lipid-lowering agents, such as statins. All individuals or their legal guardians agreed to biological sample collection procedures and signed terms of informed consent. The present research protocol was approved by the Institutional Research Board of the São Rafael Hospital (HSR protocol number: 1400535) and was conducted in compliance with the Declaration of Helsinki (1964) and its subsequent amendments. Biochemical, hematological, genetic, and immunological analyses were performed at the Clinical Analyses Laboratory of the College of Pharmaceutical Sciences, Federal University of Bahia (LACTFAR-UFBA), and at the Laboratory of Genetic Investigation and Translational Hematology at the Gonçalo Moniz Institute-FIOCRUZ (LIGHT-IGM/FIOCRUZ).

### 2.2. Clinical Manifestations

All legal guardians of the individuals with SCD were asked to complete a questionnaire containing information on clinical data regarding the occurrence of previous clinical manifestations. All information provided was confirmed by individual patient medical records.

### 2.3. Hematological and Biochemical Parameters

Blood samples were collected by HEMOBA staff following a fasting period of no less than 12 hours.

Hematological parameters were determined using a Beckman Coulter LH 780 Hematology Analyzer (Beckman Coulter, Brea, California, USA), and hemoglobin profile was confirmed by high-performance liquid chromatography using an HPLC/Variant-II hemoglobin testing system (Bio-Rad, Hercules, California, USA).

An automated A25 chemistry analyzer (Biosystems S.A, Barcelona, Catalunya, Spain) was used to determine biochemical parameters, including total bilirubin and fractions, lactate dehydrogenase (LDH), total protein and fractions, iron, hepatic metabolism, and renal profile. Ferritin levels were measured using an Access 2 Immunochemistry System (Beckman Coulter Inc., Pasadena, California, USA). C-reactive protein (CRP) and alpha-1 antitrypsin (AAT) levels were measured using an IMMAGE® Immunochemistry System (Beckman Coulter Inc., Pasadena, California, USA).

### 2.4. Lipid Profile

Total cholesterol (TC), high-density lipoprotein cholesterol (HDL-C), and triglyceride levels were determined using an A25 chemistry analyzer (Biosystems S.A, Barcelona, Catalunya, Spain), while LDL-C and VLDL-C levels were determined by the Friedewald equation [26]. TC/HDL-C, LDL-C/HDL-C, and triglyceride/HDL-C ratios were calculated to evaluate cardiovascular risk [27–31]. In addition, non-HDL-C was calculated by TC–HDL-C [32].

### 2.5. Genotype Analysis

A QIAamp DNA Blood Mini Kit (QIAGEN, Hilden, Westphalia, Germany) was used to extract genomic DNA from peripheral blood in accordance with the manufacturer's recommendations. Genotyping of *TGFBR3* polymorphisms (rs1805110, rs2038931, rs2765888, rs284157, rs284875, and rs7526590) was performed by TaqMan SNP Genotyping Assays (Applied Biosystems, Foster City, CA) on a 7500 Fast Real-Time PCR System (Applied Biosystems, Foster City, CA).

### 2.6. Linkage Disequilibrium Analysis

Haploview software (version 4.2) was used to calculate the linkage disequilibrium (LD) between each pairwise combination of SNPs and haplotype frequencies [33].

### 2.7. Statistical Analysis

The Statistical Package for the Social Sciences (SPSS) v. 20.0 software (IBM, Armonk, New York, USA) and GraphPad Prism version 6.0 (GraphPad Software, San Diego, California, USA) were used to perform all analyses, with *P* values < 0.05 considered significant. *χ*^2^ test and Fisher's exact test were used to evaluate associations between polymorphisms and clinical data. Hardy-Weinberg equilibrium (HWE) was assessed using the *χ*^2^ test. The Shapiro-Wilk test was used to determine the distribution of quantitative variables, followed by the Mann-Whitney *U* test or independent *t*-test to compare two numerical variables according to distribution.

## 3. Results

### 3.1. Baseline Characteristics of Individuals with SCD

The baseline laboratory parameters of 175 individuals with SCD, expressed as means ± standard deviation, are shown in Supplementary Table 1. Baseline laboratory characteristics showed that SCD individuals presented anemia, hemolysis, and leukocytosis, together with decreased levels of HDL-C.

### 3.2. Linkage Disequilibrium and Haplotype Analysis

Genotype frequency testing revealed all six SNPs to be in HWE (Table 1).

**Figure 1:**
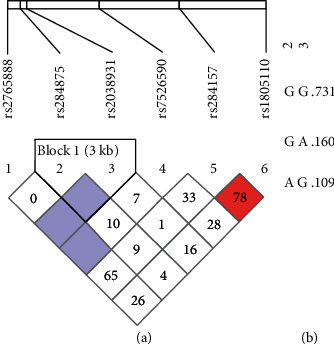
Linkage disequilibrium analysis of rs2765888 (C > T), rs284875 (A > G), rs2038931 (G > A), rs7526590 (A > T), rs284157 (C > T), and rs1805110 (G > A) using the four-gamete rule method. (a) According to HaploView 4.2 color schemes, white corresponds to *D*′ < 1 and LOD < 2, shades of pink/red indicate *D*′ < 1 and LOD ≥ 2, and light blue indicates complete LD with *D*′ = 1 and LOD < 2. (b) Identified haplotypes and associated frequencies. LOD: Log of odds ratio, a measure of confidence of the value of *D*′.

**Figure 2:**
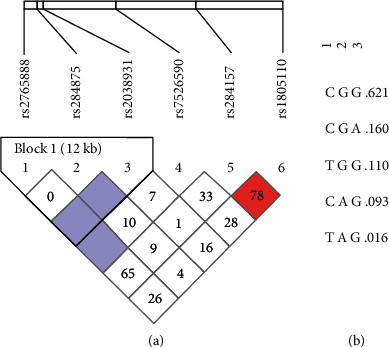
Linkage disequilibrium analysis of rs2765888 (C > T), rs284875 (A > G), rs2038931 (G > A), rs7526590 (A > T), rs284157 (C > T), and rs1805110 (G > A) using the solid spine of LD method. (a) According to HaploView 4.2 color schemes, white corresponds to *D*′ < 1 and LOD < 2, shades of pink/red indicate *D*′ < 1 and LOD ≥ 2, and light blue indicates complete LD with *D*′ = 1 and LOD < 2. (b) Identified haplotypes and associated frequencies. LOD: Log of odds ratio, a measure of confidence in the value of *D*′.

Haploview software indicated that all six SNPs met the qualifications for LD analysis, which was performed using the four-gamete rule and solid spine of LD methods. The four-gamete testing method created one block with two SNPs (rs284875 and rs2038931) showing complete LD with *D*′ = 1 and the logarithm of odds ratio (LOD) < 2 (Figure 1(a)). The haplotype evaluation of this block revealed a higher frequency of the GG haplotype (*f* = 0.731) than the others (GA = 0.160 and AG = 0.109) (Figure 1(b)). LD analysis using the solid spine of LD method created one block with three SNPs (rs2765888, rs284875, and rs2038931) (Figure 2(a)). Haplotype analysis of this block showed a higher frequency of the CGG haplotype (*f* = 0.621) than the others identified (CGA = 0.160, TGG = 0.110, CAG = 0.093, and TAG = 0.016) (Figure 2(b)).

### 3.3. Association of *TGFBR3* rs2765888, rs284875, and rs2038931 Polymorphisms with Laboratory Parameters and Clinical Manifestations in SCD

The dominant genetic model was employed in rs2765888 and rs2038931 polymorphisms, while the recessive genetic model was employed in rs284875 polymorphism to evaluate associations between alleles and laboratory biomarkers. With regard to *TGFBR3* rs2765888, individuals with CC genotype presented higher mean corpuscular hemoglobin concentration (MCHC) (*P* = 0.039) and plateletcrit (*P* = 0.027) than those with T_ genotypes (Table 2).

Regarding the *TGFBR3 rs284875* polymorphism, individuals with GG genotype presented decreased triglycerides/HDL-C ratio (*P* = 0.022) than those with A_ genotypes (Table 3).

When the *TGFBR3 rs2038931* polymorphism was evaluated individuals with GG genotype presented increased VLDL-C (*P* = 0.007), triglycerides (*P* = 0.006), TC/HDL-C ratio (*P* = 0.035), triglycerides/HDL-C ratio (*P* = 0.006), LDL-C/HDL-C ratio (*P* = 0.033), and total protein (*P* = 0.030) than those with A_ genotypes (Table 4).

Regarding clinical manifestations, no significant association was found to rs2765888 and rs284875 polymorphisms, while the A_ genotypes of rs2038931 polymorphism was associated with a previous history of cholelithiasis (*P* = 0.038) (Table 5).

### 3.4. Association of GG Haplotype with Laboratory Parameters and Clinical Manifestations in SCD

**Figure 3:**
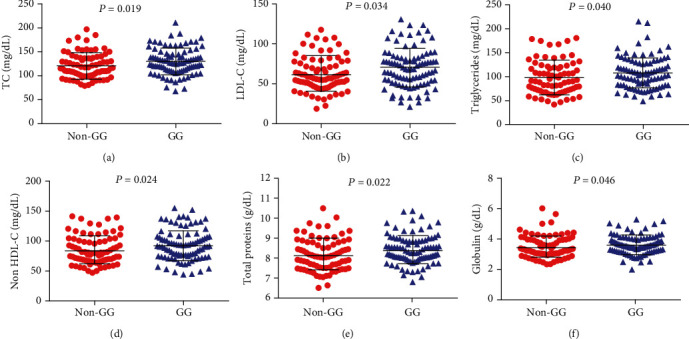
Associations between the GG haplotype and laboratory parameters in individuals with SCD. Carriers of the GG haplotype presented increased (a) total cholesterol (TC) levels, (b) low-density lipoproteins cholesterol (LDL-C) levels, (c) triglycerides, and (d) nonlow-density lipoprotein cholesterol (non-HDL-C) (all *P* values obtained by the Mann-Whitney *U* test). Carriers of the GG haplotype also had presented increased (e) total protein levels (*P* value obtained with *t*-testing) and (f) increased globulin levels (*P* value obtained by the Mann-Whitney *U* test).

**Figure 4:**
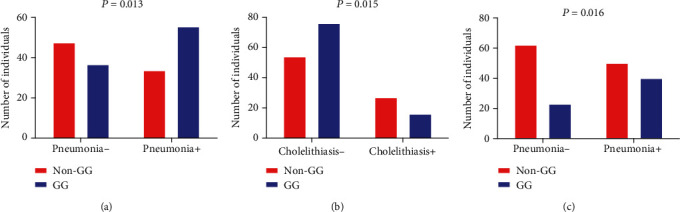
Associations between the GG and CGG haplotypes and clinical manifestations in individuals with SCD. GG haplotype was associated with (a) a previous history of pneumonia, while non-GG haplotypes were associated with (b) a previous history of cholelithiasis. The CCG haplotype was associated with (c) a previous history of pneumonia (all *P* values obtained by the Chi-square test).

A comparison of the hematological, biochemical, and immunological parameters between the GG and non-GG-haplotypes revealed that individuals with the GG haplotype presented higher total cholesterol (TC) (*P* = 0.019), low-density lipoprotein cholesterol (LDL-C) (*P* = 0.034), triglycerides (*P* = 0.040), non-HDL-C (*P* = 0.022), total proteins (*P* = 0.022), and globulin levels (*P* = 0.046) than those with non-GG haplotypes, although statistically higher, serum lipid levels in the GG haplotype are within the normal clinical range (Figure 3) (Table 6). Regarding clinical manifestations, the GG haplotype was associated with a previous history of pneumonia (*P* = 0.015) (Figure 4(a)), while non-GG haplotypes were associated with a previous history of cholelithiasis (*P* = 0.021) (Figure 4(b)) (Table 5).

### 3.5. Association of CGG Haplotype with Laboratory Parameters and Clinical Manifestations in SCD

**Figure 5:**
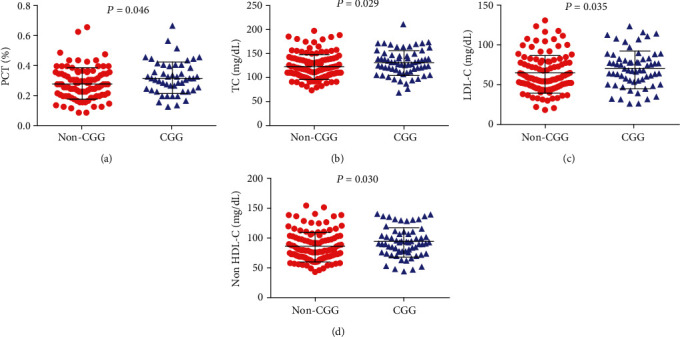
Associations between the CGG haplotype and laboratory parameters in individuals with SCD. Carriers of the CGG haplotype presented increased levels of (a) plateletcrit (PCT), (b) total cholesterol (TC), (c) low-density lipoprotein cholesterol (LDL-C), and (d) nonhigh-density lipoprotein cholesterol (non-HDL-C) (*P* values obtained by the Mann-Whitney *U* test).

A comparison of hematological, biochemical, and immunological laboratory parameters between CGG and non-CGG-haplotypes showed that individuals with CGG haplotype presented increased plateletcrit (PCT) (*P* = 0.046), TC (*P* = 0.029), LDL-C (*P* = 0.035), and non-HDL-C (*P* = 0.030) levels, although statistically higher, serum lipid levels in the CGG haplotype are within the normal clinical range (Figure 5) (Table 7). In addition, the CCG haplotype was associated with a previous history of pneumonia (*P* = 0.018) (Figure 4(c)) (Table 5).

### 3.6. Association of Clinical Manifestations with Laboratory Parameters in SCD

Once the GG and CCG haplotypes were associated with a previous history of pneumonia and the non-GG haplotype was associated with a previous history of cholelithiasis, the association between these clinical manifestations and laboratory parameters was investigated. A comparison of the hematological, biochemical, and immunological parameters between the individuals with and without previous history of pneumonia revealed that individuals with previous history of pneumonia presented higher TC (*P* = 0.004), LDL-C (*P* = 0.025), non-HDL-C (*P* = 0.012), and CRP (*P* < 0.001), as well as decreased creatinine levels (*P* = 0.014) than those without previous history of pneumonia (Table 8).

With regard to cholelithiasis, a comparison of the hematological, biochemical, and immunological parameters between the individuals with and without previous history of this clinical manifestation revealed that individuals with previous history of cholelithiasis presented increased fetal hemoglobin (*P* < 0.001), S hemoglobin (*P* = 0.002), MCV (*P* < 0.001), MCHC (*P* < 0.001), total bilirubin (*P* = 0.016), and indirect bilirubin (*P* = 0.010), as well as decreased red blood cell counts (*P* = 0.003), TC (*P* = 0.012), LDL-C (*P* = 0.003), non-HDL-C (*P* = 0.007), TC/HDL-C ratio (*P* < 0.001), LDL-C/HDL-C ratio (*P* < 0.001), and urea levels (*P* = 0.002) than those without previous history of cholelithiasis (Table 9).

## 4. Discussion

Although polymorphisms in the *TGFBR3* gene, when evaluated individually, have been associated with several clinical manifestations in SCD [14, 20–24, 34, 35], Kim et al. (2010) found that, in asthmatic individuals, these polymorphisms are more informative when evaluated in the context of haplotype, versus on an individual basis [36]. Accordingly, this study evaluated associations between *TGFBR3* haplotypes and laboratory markers, as well as clinical manifestations, in individuals with SCD.

Laboratory parameters of SCD individuals presented classical markers of hemolysis, anemia, increased leukocyte counts, as well as decreased HDL-C levels. These findings are consistent with previous reports describing SCD individuals [37–40].

In our study, six polymorphisms were evaluated; the polymorphisms rs7526590, rs284875, rs2038931, rs7526590, and rs284157 are intronic variants. Intronic variants might affect alternative splicing of the mRNA; however, until this moment, there are no evidences of association between these polymorphisms and TGFBR3 function or TGFbeta signaling. We reinforce that all the selected SNPs were previously studied and associated with the occurrence of clinical manifestation in SCD [20–24]. With regard to rs1805110 polymorphism, this is a missense variant that causes a change in the polypeptide chain where a *S* (Ser) > *F* (Phe). This polymorphism has been associated with Behçet's disease, susceptibility in HBV-related hepatocellular carcinoma, pulmonary emphysema, and aneurysm; however, it was not previously investigated in SCD [41–44].

Our results showed that individuals with the GG haplotype of *TGFBR3* had higher levels of TC, LDL-C, triglycerides, and non-HDL-C than those with non-GG haplotypes. In addition, individuals with the CGG haplotype of *TGFBR3* also presented lipid profile alterations, i.e., higher TC, LDL-C, and non-HDL-C levels than those with non-CGG haplotypes. Importantly, even statistically higher, serum lipid levels in the GG and CGG haplotypes were within the normal clinical range. The individual evaluation of polymorphisms showed that individuals with GG genotype of rs2038931 polymorphism presented increased VLDL-C, triglycerides, TC/HDL-C ratio, triglycerides/HDL-C ratio, and LDL-C/HDL-C ratio than those with A_ genotypes. Moreover, the CC genotype of rs2765888 polymorphism was also associated with increased triglycerides/HDL-C ratio. Indeed, when the *TGFBR3* rs2765888, rs284875, and rs2038931 polymorphisms, which compose the haplotypes, were individually investigated, none of them seems to contribute more than the other polymorphism to the associations found by the haplotypes. Thus, the associations between the haplotypes and lipid parameters were equally modulated by the polymorphisms. Despite the rs2038931 polymorphism has been associated with lipid markers, they were different from those identified by haplotypes.

High total cholesterol, as well as LDL-C levels, triggers the process of atherosclerosis by stimulating cholesterol accumulation and promotes an inflammatory response in the artery wall [45]. The pathophysiological mechanisms present in atherosclerosis are analogous to those identified in SCA vasculopathy, marked by enhanced oxidative stress, low NO bioavailability in addition to endothelial dysfunction; however, it is important to emphasize that in SCD there is no formation of atheroma plaques [4, 46]. Vasculopathy is responsible for several clinical manifestations in SCD, such as stroke, priapism, pulmonary hypertension, and leg ulcers [4].

Moreover, high triglyceride and non-HDL-C levels have also been associated to atherosclerosis. In the lipolysis of triglycerides, triglyceride-rich remnants are released in vessels contributing to increase inflammation, coagulation, and endothelial dysfunction [47]. Increased triglyceride levels were shown to be a potential risk factor for pulmonary hypertension, a frequent respiratory complication in individuals with SCD [29].

Non-HDL-C is a cholesterol carried by apolipoprotein B, including those carried by LDL-C and VLDL-C, and is considered a useful marker of atherosclerosis [48]. Previous studies performed in individuals with cardiovascular disease reported high non-HDL-C levels [49, 50]; however, no significant alterations in non-HDL-C levels were found in SCD individuals [32].

TC, LDL-C, triglycerides, and non-HDL-C are markers of the lipid metabolism associated with inflammation related to vascular response [45, 46, 48]. The TGF-*β* pathway has been associated with vascular inflammatory responses in several diseases. In many cases, cholesterol uptake and trafficking are also responsible for vessel wall modifications, which can contribute to inflammation [51, 52]. A previous study reported that TGF-*β* signaling regulates lipid metabolism through the induction of lipogenesis genes resulting in increased triglyceride synthesis and lipid accumulation. TGF-*β* signaling activates SMAD proteins, triggering the activation of these pathways. The inhibition of these pathways suppresses changes in gene expression associated with lipid metabolism [53]. Therefore, the identification of markers, which may modulate the endothelial inflammatory response in SCD is relevant to monitor the clinical course and may contribute to understand the disease pathophysiology.

TGFBRIII, encoded by the *TGFBR3* gene, plays an important role in regulating and mediating the signal transduction of TGF-*β* [54]. In the literature, polymorphisms in *TGFBR3* have been associated with inflammatory diseases other than SCD, such as Marfan syndrome, bladder cancer, and Behçet's disease [14, 16–18]. Marfan syndrome presents as clinical complications in the cardiovascular, skeletal, pulmonary, ocular, and nervous system; its physiopathology includes alterations in extracellular matrix deposition in vessels, similar to findings reported in SCD [16]. Behçet's disease is characterized by multisystemic vasculitis with marked inflammatory lesions in the central nervous system, skin, joints, orogenital mucosa, and eyes; analogous to SCD, the inflammatory component is the main physiopathological feature of this disease [18].

Individuals with the GG haplotype presented high levels of total proteins and globulin, which is similar to previous reports in individuals with SCD compared to controls [55, 56]. This hyperproteinemia arises from hyperglobulinemia, which is known to occur in SCD individuals as a result of erythrocyte destruction during sickling [57]. Thus, it is expected that individuals with the GG haplotype would present more prominent hemolysis than those with non-GG haplotypes. High levels of total protein were also identified in individuals with GG genotype of rs2038931 polymorphism when compared to A_ genotypes.

With regard to clinical manifestations, the haplotypes have showed to be more informative than the polymorphisms individually analyzed; both the GG and CGG haplotypes of gene *TGFBR3* were associated with the occurrence of pneumonia. In a large cohort of SCD individuals, pneumonia was one of the leading causes of hospitalization, second only to sickle cell crisis [58]. A previous study performed in individuals with SCA, the majority of whom presented *Streptococcus pneumoniae* infection, identified that a polymorphism in *TGFBR3* was associated with increased susceptibility to bacteremia [34]. *Streptococcus pneumoniae* infection is one of the main causes of pneumonia in individuals with SCD [34]. It is important to note that all individuals in this study were immunized against pneumococcal disease; therefore, it is possible that these children could have been undergoing a process of autosplenectomy, which consequently increases susceptibility to serious infections [59].

Other respiratory complications, such as asthma and bronchopulmonary dysplasia, have also been associated with *TGFBR*3 in the literature [36, 60]. Similarities exist in the pathogenesis of asthma, bronchopulmonary dysplasia, and SCD, which is marked by inflammation and the activation of several cytokines, including TGF-*β* [36, 60].

We found that individuals with a previous history of pneumonia presented increased TC, LDL-C, non-HDL-C, CRP levels, and decreased creatinine. A previous study performed in individuals with SCD identified that the occurrence of pneumonia is related to increased frequency of other respiratory complications, such as acute chest syndrome (ACS), arising from fat embolism and pathogenic infection [61]. In a cohort without SCD, high cholesterol levels were associated with death related to a miscellaneous of respiratory diseases in a large study involving adults [62].

Serum CRP levels are clinically used to differentiate pneumonia from other acute respiratory infections [63]. In SCD, community-acquired pneumonia triggers inflammatory response and lung injury [64]. Alterations in CRP levels and other immunological markers were found in SCA individuals with lung dysfunction deriving from ACS [65]. High creatinine levels were also described as a severity marker of community-acquired pneumonia; however, in our results, the individuals with SCD and previous history of pneumonia presented lower creatinine levels [66].

We also found that non-GG haplotypes were associated with the occurrence of cholelithiasis, one of the most frequent SCD complications that occurs in 26-58% of patients. In addition, an individual with a previous history of cholelithiasis presented more prominent hemolysis than those without history. This is often associated with increased bilirubin levels, and consequently with hemolysis, and has been described as part of the hemolysis-endothelial dysfunction subphenotype [4, 67, 68]. Cholelithiasis, a clinical manifestation of SCD related to chronic hemolysis, triggers bilirubin production, leading to the formation of gallstones [69]. The upregulation of the TGF-*β* pathway was previously detected in the gallbladders of individuals with cholelithiasis [70]. Moreover, an investigation of gene expression in patients undergoing cholecystectomy found significant expression of TGF-*β* receptor (TGF*β*R) I and TGF*β*RII during chronic cholelithiasis in comparison to acute cholelithiasis. However, no significant increase in TGF*β*RIII expression was found [71].

Altogether, our results suggest that *TGFBR3* haplotypes seem to be related to inflammation and the occurrence of pneumonia. Inflammation is a physiopathological mechanism present in SCD, highlighting the relevance investigating novel biomarkers of disease severity in the clinical management of individuals with SCD. To the best of our knowledge, the present study is the first attempt to demonstrate associations between *TGFBR3* haplotypes and hematological and biochemical parameters, as well as clinical manifestations in SCD.

## 5. Conclusion

Collectively, the present findings suggest that individuals with the GG and CGG haplotypes of *TGFBR3* present significant lipid profile alterations and could be associated with the occurrence of pneumonia, while the non-GG haplotypes was associated with the occurrence of cholelithiasis. Further studies are essential to evaluate *TGFBR3* haplotypes as prognostic markers and identify possible therapeutic targets in SCD individuals.

## Figures and Tables

**Table 1:** Hardy-Weinberg equilibrium values for each *TGFBR3* polymorphism.

Polymorphism	Frequencies	*χ* ^2^	*P* value
rs1805110 (G > A)			
GG	129/175	0.680	0.409
AG	44/175		
AA	2/175		
rs2038931 (G > A)			
GG	120/175	3.831	0.050
AG	54/175		
AA	1/175		
rs2765888 (C > T)			
CC	133/175	0.277	0.598
CT	40/175		
TT	2/175		
rs284157 (C > T)			
CC	73/175	0.041	0.838
CT	81/175		
TT	21/175		
rs284875 (A > G)			
AA	4/175	2.289	0.130
AG	30/175		
GG	141/175		
rs7526590 (A > T)			
AA	118/175	1.300	0.254
AT	54/175		
T	3/175		

*χ* ^2^: chi-square test.

**Table 2:** Association of *TGFBR3* rs2765888 polymorphism with laboratory biomarkers using the dominant genetic model.

Parameter	*TGFBR3* rs2765888 polymorphism		*P* value
	CC *N* = 133	T_ *N* = 42	
	Median (IQR)	Median (IQR)	
Hemoglobin pattern			
Fetal hemoglobin, %	5.70 (1.70–10.78)	4.00 (1.27–8.60)	0.217
S hemoglobin, %	80.90 (54.70–90.25)	76.20 (52.80–85.58)	0.098
Hematological markers			
RBC, 10^6^/mL	2.84 (2.49–3.96)	3.29 (2.72–3.95)	0.114
Hemoglobin, g/dL	9.00 (7.90–10.80)	9.60 (8.20–11.10)	0.219
Hematocrit, %	27.00 (23.40–32.20)	28.50 (25.33–33.00)	0.215
MCV, fL	88.85 (80.65–96.33)	85.50 (79.80–94.35)	0.361
MCH, *ρ*g	30.35 (27.35–32.80)	28.65 (26.60–32.05)	0.146
MCHC, g/dL	33.90 (33.20–34.40)	33.70 (33.18–33.90)	**0.039** ^∗^
RDW, %	20.80 (18.00–24.20)	20.60 (17.43–23.10)	0.515
Reticulocyte count, /mL	138650 (94770–171380)	140025 (88680–180015)	0.793
WBC, /mL	10600 (8075–13300)	10250 (7800–13150)	0.520^∗^
Neutrophils, /mL	4687 (3384–6650)	5550 (3550–6518)	0.403
Eosinophils, /mL	315.00 (153.30–578.80)	351.00 (155.00–548.00)	0.984
Lymphocytes, /mL	3834 (2833–4601)	3164 (2348–4264)	0.050
Monocytes, /mL	900 (600–1313)	818 (500–1158)	0.122
Platelet count, x10^3^/mL	389.00 (289.00–474.00)	332.50 (257.00–422.50)	0.071
Platelet volume average, fL	7.90 (7.40–8.60)	8.10 (7.45–8.60)	0.694^∗^
Plateletcrit, %	0.31 (0.23–0.37)	0.27 (0.20–0.31)	**0.027**
Biochemical markers			
TC, mg/dL	124.00 (105.00–145.00)	121.00 (108.80–134.50)	0.633^∗^
HDL-C, mg/dL	36.00 (31.00–42.00)	36.50 (32.75–43.00)	0.789^∗^
LDL-C, mg/dL	64.00 (50.60–80.60)	59.00 (48.10–77.85)	0.638
VLDL-C, mg/dL	20.20 (14.80–25.40)	19.40 (16.50–23.20)	0.964
Triglycerides, mg/dL	101.00 (74.00–127.00)	97.00 (82.50–116.00)	0.994
Non-HDL-C, mg/dL	85.50 (70.00–104.80)	79.50 (69.75–97.75)	0.537
TC/HDL-C ratio	3.27 (2.75–4.14)	3.22 (2.66–4.16)	0.739
Triglycerides/HDL-C ratio	2.57 (1.84–3.76)	2.61 (1.88–3.46)	0.661
LDL-C/HDL-C ratio	1.76 (1.29–2.32)	1.72 (1.18–2.29)	0.828
Total bilirubin, mg/dL	2.05 (1.26–3.13)	1.82 (1.21–3.42)	0.704
Direct bilirubin, mg/dL	0.35 (0.26–0.50)	0.35 (0.24–0.43)	0.662
Indirect bilirubin, mg/dL	1.63 (0.89–2.74)	1.41 (0.91–3.12)	0.924
LDH, U/L	868.00 (654.00–1239.00)	847.50 (610.80–1265.00)	0.593
ALT, U/L	15.00 (11.00–19.00)	12.00 (9.00–20.00)	0.213
AST, U/L	38.00 (26.00–51.25)	34.50 (22.75–56.75)	0.418
Total protein, g/dL	8.19 (7.72–8.84)	8.27 (7.93–8.90)	0.469
Albumin, g/dL	4.75 (4.57–4.95)	4.86 (4.65–5.02)	0.237^∗^
Globulin, g/dL	3.48 (3.03–4.00)	3.42 (3.14–3.94)	0.912
Albumin/globulin ratio	1.38 (1.21–1.58)	1.37 (1.25–1.61)	0.679^∗^
Iron, mcg/dL	92.00 (73.75–117.00)	93.00 (62.00–134.50)	0.762
Ferritin, *η*g/mL	147.50 (90.68–243.80)	121.20 (45.10–169.80)	0.221
Urea nitrogen, mg/dL	17.00 (13.96–21.00)	18.02 (14.31–21.82)	0.201
Creatinine, mg/dL	0.48 (0.38–0.58)	0.51 (0.39–0.68)	0.147
CRP, mg/L	2.61 (1.73–3.78)	2.39 (1.81–5.89)	0.255
AAT, mg/dL	69.30 (37.60–121.30)	72.00 (38.00–91.30)	0.886

RBC: red blood cells; MCV: mean cell volume; MCH: mean cell hemoglobin; MCHC: mean corpuscular hemoglobin concentration; RDW: red cell distribution width; LDH: lactate dehydrogenase; WBC: white blood cells; TC: total cholesterol; HDL-C: high-density lipoprotein cholesterol; LDL-C: low-density lipoprotein cholesterol; VLDL-C: very low-density lipoprotein cholesterol; AST: aspartate aminotransferase; ALT: alanine aminotransferase; CRP: C reactive protein; AAT: alpha 1-antitrypsin; IQR: interquartile range. Bold values indicate significance at *P* < 0.05. All *P* values obtained by the Mann–Whitney *U* test, except for those with asterisk (^∗^), for which the independent *t*-test was used.

**Table 3:** Association of *TGFBR3* rs284875 polymorphism with laboratory biomarkers using the recessive genetic model.

Parameter	*TGFBR3* rs284875 polymorphism		*P* value
	GG *N* = 141	A_ *N* = 34	
	Median (IQR)	Median (IQR)	
Hemoglobin pattern			
Fetal hemoglobin, %	4.70 (1.30–10.30)	6.20 (2.30–9.80)	0.344
S hemoglobin, %	79.90 (52.00–88.70)	83.55 (56.35–90.13)	0.239
Hematological markers			
RBC, 10^6^/mL	2.98 (2.59–3.98)	2.63 (2.40–3.68)	0.090
Hemoglobin, g/dL	9.10 (8.10–11.10)	8.35 (7.77–10.35)	0.183
Hematocrit, %	27.50 (24.00–32.60)	25.70 (22.60–31.05)	0.141
MCV, fL	87.10 (80.30–94.80)	90.35 (82.03–98.30)	0.151^∗^
MCH, *ρ*g	29.55 (27.20–32.40)	31.15 (27.60–34.10)	0.152^∗^
MCHC, g/dL	33.70 (33.20–34.20)	34.00 (33.28–34.53)	0.193
RDW, %	20.70 (17.90–23.50)	21.70 (17.78–25.45)	0.350
Reticulocyte count, /mL	138650 (94770–179340)	136010 (87075–184758)	0.605
WBC, /mL	10750 (8000–13225)	10100 (7875–13525)	0.489
Neutrophils, /mL	5060 (3461–6600)	4348 (3386–6677)	0.413
Eosinophils, /mL	309.50 (153.80–546.00)	396.50 (162.50–613.50)	0.671
Lymphocytes, /mL	3629 (2723–4508)	3795 (2375–4656)	0.825
Monocytes, /mL	900 (600–1296)	810 (560–1140)	0.686
Platelet count, x10^3^/mL	376.00 (278.00–467.00)	388.50 (273.30–457.00)	0.909^∗^
Platelet volume average, fL	8.10 (7.40–8.60)	7.80 (7.37–8.42)	0.256^∗^
Plateletcrit, %	0.29 (0.23–0.36)	0.29 (0.19–0.36)	0.704
Biochemical markers			
TC, mg/dL	123.00 (108.00–140.00)	118.00 (97.00–140.50)	0.296
HDL-C, mg/dL	37.00 (32.00–43.00)	33.00 (29.00–41.00)	0.180^∗^
LDL-C, mg/dL	64.60 (51.55–80.35)	55.20 (44.30–75.30)	0.120
VLDL-C, mg/dL	19.40 (14.60–23.60)	21.00 (16.00–27.60)	0.091
Triglycerides, mg/dL	97.00 (73.00–118.00)	105.00 (80.00–138.00)	0.098
Non-HDL-C, mg/dL	85.00 (72.00–103.00)	81.00 (64.50–107.00)	0.489
TC/HDL-C ratio	3.24 (2.76–4.15)	3.29 (2.73–4.50)	0.629
Triglycerides/HDL-C ratio	2.48 (1.82–3.50)	2.93 (2.31–4.30)	**0.022**
LDL-C/HDL-C ratio	1.75 (1.33–2.34)	1.78 (1.21–2.20)	0.877
Total bilirubin, mg/dL	2.04 (1.24–3.23)	2.00 (1.40–3.06)	0.786
Direct bilirubin, mg/dL	0.35 (0.24–0.43)	0.39 (0.29–0.54)	0.072
Indirect bilirubin, mg/dL	1.58 (0.89–2.90)	1.48 (1.01–2.78)	0.886
LDH, U/L	856.00 (630.00–1217.00)	954.00 (614.50–1354.00)	0.614
ALT, U/L	14.00 (10.00–19.00)	15.00 (9.25–19.50)	0.841
AST, U/L	37.00 (26.00–50.00)	41.00 (25.50–57.00)	0.899
Total protein, g/dL	8.22 (7.85–8.85)	8.09 (7.51–8.89)	0.427
Albumin, g/dL	4.76 (4.60–4.98)	4.88 (4.50–4.99)	0.858
Globulin, g/dL	3.47 (3.11–3.96)	3.25 (2.98–4.11)	0.437
Albumin/globulin ratio	1.36 (1.22–1.58)	1.42 (1.22–1.61)	0.442^∗^
Iron, mcg/dL	92.00 (72.00–120.00)	92.00 (72.50–126.00)	0.921
Ferritin, *η*g/mL	145.70 (91.63–214.90)	239.60 (50.93–524.70)	0.272
Urea nitrogen, mg/dL	17.00 (14.00–21.00)	17.99 (14.11–21.07)	0.996
Creatinine, mg/dL	0.48 (0.38–0.60)	0.50 (0.34–0.64)	0.962
CRP, mg/L	2.39 (1.71–3.57)	2.66 (1.87–4.82)	0.240
AAT, mg/dL	71.30 (37.10–113.00)	69.00 (45.20–131.30)	0.444

RBC: red blood cells; MCV: mean cell volume; MCH: mean cell hemoglobin; MCHC: mean corpuscular hemoglobin concentration; RDW: red cell distribution width; LDH: lactate dehydrogenase; WBC: white blood cells; TC: total cholesterol; HDL-C: high-density lipoprotein cholesterol; LDL-C: low-density lipoprotein cholesterol; VLDL-C: very low-density lipoprotein cholesterol; AST: aspartate aminotransferase; ALT: alanine aminotransferase; CRP: C reactive protein; AAT: alpha 1-antitrypsin; IQR: interquartile range. Bold values indicate significance at *P* < 0.05. All *P* values obtained by the Mann–Whitney *U* test, except for those with asterisk (^∗^), for which the independent *t*-test was used.

**Table 4:** Association of *TGFBR3* rs2038931 polymorphism with laboratory biomarkers using the dominant genetic model.

Parameter	*TGFBR3* rs2038931 polymorphism		*P* value
	GG *N* = 120	A_ *N* = 55	
	Median (IQR)	Median (IQR)	
Hemoglobin pattern			
Fetal hemoglobin, %	4.80 (1.70–8.75)	5.50 (1.30–12.63)	0.486
S hemoglobin, %	80.30 (54.50–89.90)	79.95 (52.15–87.60)	0.802
Hematological markers			
RBC, 10^6^/mL	2.96 (2.51–3.97)	2.95 (2.56–3.79)	0.978
Hemoglobin, g/dL	9.00 (7.90–10.88)	9.20 (8.10–10.80)	0.510
Hematocrit, %	27.00 (23.30–32.65)	27.50 (24.30–32.10)	0.524
MCV, fL	87.30 (80.10–94.30)	91.20 (80.80–97.30)	0.177^∗^
MCH, *ρ*g	29.60 (27.15–32.65)	30.90 (27.60–32.60)	0.270^∗^
MCHC, g/dL	33.80 (33.20–34.40)	33.70 (33.20–34.30)	0.393^∗^
RDW, %	21.30 (18.00–24.40)	20.00 (17.60–23.00)	0.074
Reticulocyte count, /mL	131330 (88245–174570)	144690 (98880–180540)	0.070^∗^
WBC, /mL	10700 (8150–13025)	10450 (7550–13625)	0.349
Neutrophils, /mL	5078 (3478–6600)	4402 (3356–6675)	0.270
Eosinophils, /mL	300.00 (160.00–544.00)	326.00 (142.00–590.00)	0.996
Lymphocytes, /mL	3702 (2783–4601)	3493 (2666–4454)	0.339
Monocytes, /mL	894 (600–1297)	800 (552–1288)	0.499
Platelet count, x10^3^/mL	383.00 (278.00–457.00)	372.00 (274.00–467.00)	0.951
Platelet volume average, fL	7.90 (7.50–8.52)	8.10 (7.30–8.70)	0.278^∗^
Plateletcrit, %	0.29 (0.23–0.36)	0.29 (0.22–0.36)	0.877
Biochemical markers			
TC, mg/dL	126.50 (109.30–141.80)	116.00 (103.00–137.50)	0.116
HDL-C, mg/dL	36.00 (32.00–42.00)	37.00 (32.00–45.00)	0.121
LDL-C, mg/dL	64.40 (50.60–81.40)	58.60 (49.90–78.60)	0.236
VLDL-C, mg/dL	20.50 (16.15–25.65)	17.40 (12.35–23.20)	**0.007**
Triglycerides, mg/dL	103.00 (81.00–129.00)	88.00 (62.00–116.00)	**0.006**
Non-HDL-C, mg/dL	90.00 (71.00–106.00)	79.00 (67.00–98.50)	0.068
TC/HDL-C ratio	3.33 (2.77–4.35)	3.10 (2.67–3.57)	**0.035**
Triglycerides/HDL-C ratio	2.69 (1.97–3.73)	2.32 (1.53–3.10)	**0.006**
LDL-C/HDL-C ratio	1.81 (1.34–2.43)	1.58 (1.15–2.00)	**0.033**
Total bilirubin, mg/dL	2.07 (1.31–3.06)	1.76 (1.15–3.52)	0.654
Direct bilirubin, mg/dL	0.36 (0.24–0.49)	0.35 (0.28–0.43)	0.966
Indirect bilirubin, mg/dL	1.60 (0.98–2.63)	1.29 (0.85–3.12)	0.695
LDH, U/L	907.50 (630.50–1318.00)	841.00 (609.00–1085.00)	0.227
ALT, U/L	14.50 (10.75–20.00)	15.00 (10.00–19.00)	0.784
AST, U/L	37.00 (26.00–54.00)	38.00 (24.00–49.00)	0.627
Total protein, g/dL	8.27 (7.93–8.87)	8.00 (7.50–8.66)	**0.030** ^∗^
Albumin, g/dL	4.83 (4.60–5.00)	4.71 (4.53–4.90)	0.066^∗^
Globulin, g/dL	3.50 (3.11–4.00)	3.30 (2.94–3.93)	0.078
Albumin/globulin ratio	1.37 (1.21–1.51)	1.40 (1.23–1.64)	0.381^∗^
Iron, mcg/dL	92.00 (72.00–117.00)	92.00 (72.50–128.30)	0.483
Ferritin, *η*g/mL	134.00 (71.55–189.10)	190.30 (89.33–263.00)	0.082
Urea nitrogen, mg/dL	17.22 (14.30–20.94)	15.00 (13.40–22.00)	0.367^∗^
Creatinine, mg/dL	0.49 (0.36–0.62)	0.48 (0.38–0.59)	0.815
CRP, mg/L	2.40 (1.71–3.79)	2.58 (1.77–3.51)	0.866
AAT, mg/dL	68.20 (38.08–117.80)	73.80 (37.30–120.00)	0.859

RBC: red blood cells; MCV: mean cell volume; MCH: mean cell hemoglobin; MCHC: mean corpuscular hemoglobin concentration; RDW: red cell distribution width; LDH: lactate dehydrogenase; WBC: white blood cells; TC: total cholesterol; HDL-C: high-density lipoprotein cholesterol; LDL-C: low-density lipoprotein cholesterol; VLDL-C: very-low-density lipoprotein cholesterol; AST: aspartate aminotransferase; ALT: alanine aminotransferase; CRP: C reactive protein; AAT: alpha 1-antitrypsin; IQR: interquartile range. Bold values indicate significance at *P* < 0.05. All *P* values obtained by the Mann–Whitney *U* test, except for those with asterisk (^∗^), for which the independent *t*-test was used.

**Table 5:** Association of *TGFBR3* rs2765888, rs284875, and rs2038931 polymorphism and *TGFBR3* haplotypes with clinical manifestations in SCD.

Clinical manifestation	Polymorphisms									Haplotypes					
	rs2765888		*P* value	rs284875		*P* value	rs2038931		*P* value	GG *N* = 93	Non-GG *N* = 82	*P* value	CGG *N* = 63	Non-CGG *N* = 112	*P* value
	CC *N* = 133	T_ *N* = 42		GG *N* = 141	A_ N =34		GG *N* = 120	A_ *N* = 55							
Acute chest syndrome	28	11	0.485	32	7	0.791	25	14	0.495	19	20	0.529	12	27	0.440
Cholelithiasis	37	6	0.075	33	10	0.465	24	19	**0.038**	16	27	**0.015**	13	30	0.364
Infections	85	28	0.744	89	24	0.413	79	34	0.606	61	52	0.763	39	74	0.580
Leg ulcer	12	6	0.327	12	6	0.115	11	7	0.471	8	10	0.434	3	15	0.117∗
Pneumonia	71	19	0.357	74	16	0.570	66	24	0.162	56	34	**0.013**	40	50	**0.016**
Painful crises	88	31	0.354	96	23	0.960	84	35	0.402	67	52	0.222	47	72	0.160
Stroke	10	4	0.745∗	12	2	0.999∗	9	5	0.767∗	7	7	0.805	4	10	0.772∗
Vaso-occlusive events	34	17	0.063	39	12	0.379	35	16	0.991	27	24	0.972	16	35	0.413

Bold values indicate significance at *P* < 0.05. All *P* values obtained by the Chi-square, except for those with asterisk (^∗^), for which the Fisher's exact test was used.

**Table 6:** Association of *TGFBR3* GG haplotype with laboratory biomarkers.

Parameter	*TGFBR3* GG haplotype		*P* value
	GG *N* = 93	Non-GG *N* = 82	
	Median (IQR)	Median (IQR)	
Hemoglobin pattern			
Fetal hemoglobin, %	80.10 (52.08–89.88)	80.20 (54.50–88.60)	0.688
S hemoglobin, %	4.50 (1.40–8.60)	6.20 (1.70–11.600)	0.142
Hematological markers			
RBC, 10^6^/mL	2.98 (2.57–3.98)	2.90 (2.49–3.71)	0.345
Hemoglobin, g/dL	9.10 (8.00–11.10)	9.10 (8.10–10.73)	0.800
Hematocrit, %	27.00 (23.90–33.00)	27.35 (23.48–32.10)	0.765
MCV, fL	86.20 (80.15–93.85)	90.70 (80.78–97.50)	0.063^∗^
MCH, *ρ*g	29.10 (27.03–32.33)	30.95 (27.55–32.85)	0.778^∗^
MCHC, g/dL	33.70 (33.20–34.20)	33.80 (33.20–34.40)	0.819
RDW, %	21.30 (18.10–24.40)	20.25 (17.70–23.70)	0.217
Reticulocyte count,/mL	133140 (90480–173360)	142055 (94085–181290)	0.478^∗^
WBC, /mL	10800 (8700–13000)	10100 (7650–13550)	0.135^∗^
Neutrophils, /mL	5282 (3800–6600)	4348 (3350–6713)	0.091
Eosinophils, /mL	300.00 (176.50–557.80)	326.00 (143.50–572.00)	0.862
Lymphocytes, /mL	3629 (2842–4600)	3700 (2626–4514)	0.591
Monocytes, /mL	900.00 (600.00–1300.00)	805.00 (563.50–1265.00)	0.322
Platelet count, x10^3^/mL	379.00 (281.00–474.00)	383.00 (271.80–462.30)	0.655^∗^
Platelet volume average, fL	8.00 (7.50–8.60)	7.95 (7.30–8.60)	0.875^∗^
Plateletcrit, %	0.29 (0.23–0.36)	0.28 (0.21–0.36)	0.375
Biochemical markers			
TC, mg/dL	127.00 (111.50–145.00)	116.50 (100.30–138.00)	**0.019**
HDL-C, mg/dL	36.00 (32.00–42.75)	36.00 (31.00–43.75)	0.798^∗^
LDL-C, mg/dL	68.40 (52.50–82.60)	57.70 (48.10–77.45)	**0.034**
VLDL-C, mg/dL	20.60 (16.20–25.40)	17.80 (13.80–23.80)	0.050
Triglycerides, mg/dL	103.50 (81.00–127.50)	89.50 (69.25–118.80)	**0.040**
Non-HDL-C, mg/dL	90.50 (74.00–106.80)	79.00 (66.25–99.75)	**0.024**
TC/HDL-C ratio	3.34 (2.83–4.26)	3.20 (2.67–3.88)	0.133
Triglycerides/HDL-C ratio	2.71 (1.93–3.73)	2.47 (1.70–3.30)	0.092
LDL-C/HDL-C ratio	1.80 (1.38–2.43)	1.64 (1.20–2.11)	0.135
Total bilirubin, mg/dL	2.07 (1.26–3.18)	1.86 (1.22–3.14)	0.573
Direct bilirubin, mg/dL	0.25 (0.23–0.45)	0.35 (0.28–0.50)	0.378
Indirect bilirubin, mg/dL	1.67 (0.98–2.90)	1.34 (0.87–2.75)	0.339
LDH, U/L	917.00 (644.50–1304.00)	852.00 (614.00–1137.00)	0.276
ALT, U/L	14.50 (11.00–20.00)	14.00 (10.00–19.00)	0.326
AST, U/L	37.50 (26.00–54.75)	37.00 (24.25–50.75)	0.535
Total protein, g/dL	8.33 (8.01–8.88)	8.09 (7.53–8.82)	**0.022** ^∗^
Albumin, g/dL	4.79 (4.60–5.05)	4.74 (4.53–4.94)	0.158
Globulin, g/dL	3.52 (3.19–4.00)	3.27 (2.98–3.94)	**0.046**
Albumin/globulin ratio	1.35 (1.20–1.50)	1.41 (1.22–1.62)	0.147^∗^
Iron, mcg/dL	92.00 (74.00–117.00)	92.00 (70.25–127.00)	0.795
Ferritin, *η*g/mL	144.60 (98.60–200.60)	177.30 (72.70–300.40)	0.440
Urea nitrogen, mg/dL	17.19 (14.81–21.00)	15.74 (13.50–21.14)	0.169
Creatinine, mg/dL	0.48 (0.38–0.61)	0.48 (0.38–0.60)	0.999
CRP, mg/L	2.41 (1.67–3.78)	2.57 (1.80–3.53)	0.730
AAT, mg/dL	71.30 (37.35–118.50)	69.05 (38.23–118.30)	0.984

RBC: red blood cells; MCV: mean cell volume; MCH: mean cell hemoglobin; MCHC: mean corpuscular hemoglobin concentration; RDW: red cell distribution width; LDH: lactate dehydrogenase; WBC: white blood cells; TC: total cholesterol; HDL-C: high-density lipoprotein cholesterol; LDL-C: low-density lipoprotein cholesterol; VLDL-C: very-low-density lipoprotein cholesterol; AST: aspartate aminotransferase; ALT: alanine aminotransferase; CRP: C reactive protein; AAT: alpha 1-antitrypsin; IQR: interquartile range. Bold values indicate significance at *P* < 0.05. All *P* values obtained by the Mann–Whitney *U* test, except for those with asterisk (∗), for which the independent *t*-test was used.

**Table 7:** Association of *TGFBR3* CGG haplotype with laboratory biomarkers.

Parameter	*TGFBR3* CGG haplotype		*P* value
	CGG *N* = 63	Non-CGG *N* = 112	
	Median (IQR)	Median (IQR)	
Hemoglobin pattern			
Fetal hemoglobin, %	4.50 (1.70–8.15)	5.85 (1.52–11.70)	0.331
S hemoglobin, %	80.70 (52.00–90.53)	79.70 (55.35–88.20)	0.676
Hematological markers			
RBC, 10^6^/mL	2.98 (2.54–3.99)	2.95 (2.53–3.78)	0.681
Hemoglobin, g/dL	9.00 (7.85–11.35)	9.15 (8.10–10.80)	0.958
Hematocrit, %	27.00 (23.70–33.50)	27.40 (23.58–32.10)	0.977
MCV, fL	86.20 (80.05–93.78)	89.75 (80.73–97.03)	0.230
MCH, *ρ*g	29.35 (27.13–32.38)	30.25 (27.40–32.68)	0.242^∗^
MCHC, g/dL	33.90 (33.20–34.45)	33.70 (33.20–34.28)	0.304^∗^
RDW, %	21.60 (18.10–24.50)	20.40 (17.70–23.70)	0.177
Reticulocyte count, /mL	131100 (106860–170440)	139385 (91350–180125)	0.948
WBC, /mL	10800 (9150–13000)	10300 (7800–13400)	0.152^∗^
Neutrophils, /mL	4876 (3750–6650)	4810 (3392–6600)	0.452
Eosinophils, /mL	300.00 (182.00–520.00)	335.00 (146.50–552.50)	0.969
Lymphocytes, /mL	3840 (2943–4640)	3500 (2574–4440)	0.090
Monocytes, /mL	944.00 (597.00–1346.00)	840.50 (575.30–1253.00)	0.209
Platelet count, x10^3^/mL	389.00 (310.50–484.50)	371.50 (267.30–454.80)	0.171^∗^
Platelet volume average, fL	7.90 (7.50–8.50)	8.00 (7.30–8.60)	0.989^∗^
Plateletcrit, %	0.32 (0.24–0.40)	0.28 (0.21–0.35)	**0.046**
Biochemical markers			
TC, mg/dL	128.00 (112.00–147.00)	119.00 (103.50–138.30)	**0.029**
HDL-C, mg/dL	36.00 (31.75–42.00)	36.00 (32.00–43.00)	0.645^∗^
LDL-C, mg/dL	68.40 (55.40–82.80)	58.50 (48.40–78.00)	**0.035**
VLDL-C, mg/dL	20.80 (15.45–26.40)	19.10 (14.45–23.50)	0.176
Triglycerides, mg/dL	105.00 (77.50–132.50)	94.00 (72.50–117.00)	0.141
Non-HDL-C, mg/dL	91.00 (76.00–108.00)	79.00 (68.00–101.50)	**0.030**
TC/HDL-C ratio	3.36 (2.86–4.32)	3.22 (2.67–3.96)	0.134
Triglycerides/HDL-C ratio	2.89 (1.87–3.96)	2.51 (1.82–3.45)	0.086
LDL-C/HDL-C ratio	1.83 (1.41–2.43)	1.68 (1.20–2.20)	0.135
Total bilirubin, mg/dL	2.08 (1.26–3.14)	1.92 (1.25–3.20)	0.657
Direct bilirubin, mg/dL	0.34 (0.23–0.45)	0.36 (0.26–0.49)	0.373
Indirect bilirubin, mg/dL	1.60 (0.96–2.66)	1.47 (0.88–2.79)	0.664
LDH, U/L	886.00 (651.00–1261.00)	855.00 (614.50–1224.00)	0.558
ALT, U/L	15.00 (11.00–18.00)	14.00 (10.00–20.00)	0.675
AST, U/L	36.50 (26.00–49.50)	38.00 (24.00–54.00)	0.855
Total protein, g/dL	8.33 (8.02–8.89)	8.09 (7.66–8.82)	0.055^∗^
Albumin, g/dL	4.77 (4.60–5.05)	4.79 (4.59–4.98)	0.625^∗^
Globulin, g/dL	3.60 (3.28–4.03)	3.32 (3.00–3.93)	**0.022**
Albumin/globulin ratio	1.31 (1.20–1.50)	1.40 (1.26–1.61)	0.059^∗^
Iron, mcg/dL	88.00 (74.25–105.00)	92.00 (67.50–127.50)	0.363
Ferritin, *η*g/mL	142.00 (98.30–219.20)	155.70 (73.25–254.30)	0.931
Urea nitrogen, mg/dL	17.00 (14.70–20.49)	17.10 (14.00–21.89)	0.915
Creatinine, mg/dL	0.48 (0.38–0.59)	0.49 (0.38–0.61)	0.605
CRP, mg/L	2.43 (1.59–3.78)	2.37 (1.80–3.53)	0.780
AAT, mg/dL	65.80 (34.75–121.00)	72.55 (39.20–116.00)	0.566

RBC: red blood cells; MCV: mean cell volume; MCH: mean cell hemoglobin; MCHC: mean corpuscular hemoglobin concentration; RDW: red cell distribution width; LDH: lactate dehydrogenase; WBC: white blood cells; TC: total cholesterol; HDL-C: high-density lipoprotein cholesterol; LDL-C: low-density lipoprotein cholesterol; VLDL-C: very-low-density lipoprotein cholesterol; AST: aspartate aminotransferase; ALT: alanine aminotransferase; CRP: C reactive protein; AAT: alpha 1-antitrypsin; IQR: interquartile range. Bold values indicate significance at *P* < 0.05. All *P* values obtained by the Mann–Whitney *U* test, except for those with asterisk (^∗^), for which the independent *t*-test was used.

**Table 8:** Association of occurrence of pneumonia with laboratory biomarkers.

Parameter	Pneumonia- *N* = 85	Pneumonia+ *N* = 90	*P* value
	Median (IQR)	Median (IQR)	
Hemoglobin pattern			
Fetal hemoglobin, %	4.90 (1.30–8.30)	6.00 (1.77–11.90)	0.208
S hemoglobin, %	76.20 (51.70–89.50)	80.95 (56.75–89.20)	0.347
Hematological markers			
RBC, 10^6^/mL	2.86 (2.53–4.10)	2.98 (2.53–3.73)	0.499
Hemoglobin, g/dL	9.00 (8.05–11.35)	9.10 (8.00–10.65)	0.685
Hematocrit, %	27.00 (23.45–33.35)	27.45 (23.93–31.95)	0.760
MCV, fL	85.60 (80.30–93.50)	89.80 (81.40–97.80)	0.194
MCH, *ρ*g	29.30 (27.20–32.45)	30.50 (27.40–33.10)	0.365^∗^
MCHC, g/dL	33.90 (33.45–34.40)	33.60 (33.10–34.30)	0.226^∗^
RDW, %	20.40 (17.40–23.75)	21.20 (18.00–24.38)	0.103
Reticulocyte count, /mL	127880 (86020–171955)	145305 (105170–183728)	0.100^∗^
WBC, /mL	10500 (7350–13050)	10600 (8300–13500)	0124^∗^
Neutrophils, /mL	4621 (3290–6468)	5060 (3600–7400)	0.172
Eosinophils, /mL	349.00 (130.00–595.50)	300.00 (179.00–553.00)	0.781
Lymphocytes, /mL	3549 (2400–4490)	3790 (2954–4537)	0.152
Monocytes, /mL	804.00 (600.00–1259.00)	915.50 (573.80–1300.00)	0.412
Platelet count, x10^3^/mL	357.00 (247.00–432.50)	395.00 (315.50–491.00)	0.051
Platelet volume average, fL	8.10 (7.35–8.70)	7.90 (7.50–8.40)	0.245∗
Plateletcrit, %	0.28 (0.20–0.35)	0.30 (0.24–0.40)	0.062
Biochemical markers			
TC, mg/dL	117.00 (102.00–138.00)	128.50 (110.50–150.80)	**0.004** ^∗^
HDL-C, mg/dL	37.00 (31.50–42.50)	35.00 (32.00–43.00)	0.979
LDL-C, mg/dL	58.80 (49.80–78.00)	68.40 (50.90–87.35)	**0.025** ^∗^
VLDL-C, mg/dL	18.50 (14.15–23.30)	20.60 (15.75–25.95)	0.084
Triglycerides, mg/dL	92.50 (70.75–116.50)	103.00 (79.50–131.30)	0.073
Non-HDL-C, mg/dL	78.00 (67.50–100.00)	91.00 (73.00–111.00)	**0.012**
TC/HDL-C ratio	3.13 (2.62–3.94)	3.28 (2.87–4.37)	0.052
Triglycerides/HDL-C ratio	2.48 (1.81–3.35)	2.71 (1.88–4.00)	0.073
LDL-C/HDL-C ratio	1.67 (1.16–2.20)	1.77 (1.40–2.43)	0.184
Total bilirubin, mg/dL	2.02 (1.16–3.06)	2.02 (1.29–3.24)	0.716
Direct bilirubin, mg/dL	0.34 (0.25–0.46)	0.37 (0.26–0.49)	0.318
Indirect bilirubin, mg/dL	1.53 (0.88–2.74)	1.60 (0.99–2.91)	0.565
LDH, U/L	866.50 (605.00–1214.00)	858.50 (665.00–1324.00)	0.326
ALT, U/L	12.50 (10.00–18.75)	15.00 (11.00–20.00)	0.161
AST, U/L	38.00 (25.25–50.75)	36.00 (26.00–53.50)	0.968
Total protein, g/dL	8.30 (7.70–8.85)	8.16 (7.85–8.85)	0.957^∗^
Albumin, g/dL	4.85 (4.60–5.02)	4.77 (4.57–4.98)	0.123^∗^
Globulin, g/dL	3.42 (3.00–4.01)	3.47 (3.13–3.94)	0.560
Albumin/globulin ratio	1.40 (1.22–1.60)	1.35 (1.21–1.50)	0.267^∗^
Iron, mcg/dL	92.00 (70.75–127.50)	92.00 (72.50–108.50)	0.231
Ferritin, *η*g/mL	151.50 (89.75–238.20)	139.30 (71.70–213.40)	0.466
Urea nitrogen, mg/dL	16.64 (14.00–21.22)	17.50 (14.39–21.00)	0.776
Creatinine, mg/dL	0.50 (0.42–0.66)	0.46 (0.36–0.57)	**0.014**
CRP, mg/L	2.12 (1.63–2.98)	3.21 (1.96–4.79)	**<0.001**
AAT, mg/dL	70.25 (39.23–114.80)	72.00 (37.10–120.00)	0.869

RBC: red blood cells; MCV: mean cell volume; MCH: mean cell hemoglobin; MCHC: mean corpuscular hemoglobin concentration; RDW: red cell distribution width; LDH: lactate dehydrogenase; WBC: white blood cells; TC: total cholesterol; HDL-C: high-density lipoprotein cholesterol; LDL-C: low-density lipoprotein cholesterol; VLDL-C: very-low-density lipoprotein cholesterol; AST: aspartate aminotransferase; ALT: alanine aminotransferase; CRP: C reactive protein; AAT: alpha 1-antitrypsin; IQR: interquartile range. Bold values indicate significance at *P* < 0.05. All *P* values obtained by the Mann–Whitney *U* test, except for those with asterisk (^∗^), for which the independent *t*-test was used.

**Table 9:** Association of occurrence of cholelithiasis with laboratory biomarkers.

Parameter	Cholelithiasis- *N* = 132	Cholelithiasis+ *N* = 43	*P* value
	Median (IQR)	Median (IQR)	
Hemoglobin pattern			
Fetal hemoglobin, %	3.50 (1.20–7.50)	7.50 (3.60–13.75)	**<0.001**
S hemoglobin, %	78.70 (51.93–89.35)	85.40 (78.35–90.45)	**0.002**
Hematological markers			
RBC, 10^6^/mL	3.11 (2.59–3.97)	2.63 (2.33–3.34)	**0.003**
Hemoglobin, g/dL	9.25 (8.10–11.03)	8.60 (7.60–10.50)	0.130
Hematocrit, %	27.50 (24.00–32.55)	26.00 (22.70–31.80)	0.176
MCV, fL	85.60 (79.80–93.30)	94.30 (88.40–104.80)	**<0.001** ^∗^
MCH, *ρ*g	28.80 (26.80–31.80)	32.30 (30.10–35.53)	**<0.001** ^∗^
MCHC, g/dL	33.80 (33.23–34.40)	33.70 (33.10–34.10)	0.458^∗^
RDW, %	20.80 (17.73–24.15)	20.80 (18.10–24.00)	0.999
Reticulocyte count, /mL	142285 (99920–142285)	123200 (79580–157800)	0.085
WBC, /mL	10800 (8100–13300)	9900 (7400–13200)	0.277^∗^
Neutrophils, /mL	5026 (3600–6600)	4200 (3080–6600)	0.096
Eosinophils, /mL	345.00 (163.00–597.00)	288.00 (100.00–444.00)	0.097
Lymphocytes, /mL	3658 (2800–4532)	3800 (2652–4700)	0.626
Monocytes, /mL	915.50 (600.00–1300.00)	784.00 (500.00–1260.00)	0.224
Platelet count, x10^3^/mL	390.00 (289.30–476.00)	352.00 (238.00–462.00)	0.149
Platelet volume average, fL	8.10 (7.50–8.60)	7.90 (7.20–8.60)	0.173^∗^
Plateletcrit, %	0.30 (0.23–0.36)	0.28 (0.20–0.32)	0.104
Biochemical markers			
TC, mg/dL	126.00 (110.00–145.00)	110.00 (97.00–132.00)	**0.012** ^∗^
HDL-C, mg/dL	36.00 (32.00–43.00)	36.00 (32.00–44.00)	0.796^∗^
LDL-C, mg/dL	64.70 (52.15–83.15)	53.80 (45.60–75.60)	**0.003**
VLDL-C, mg/dL	20.20 (15.35–23.90)	17.50 (14.45–25.20)	0.399
Triglycerides, mg/dL	101.00 (77.00–121.00)	88.00 (72.50–125.00)	0.355
Non-HDL-C, mg/dL	90.00 (72.00–106.00)	74.00 (65.00–91.00)	**0.007**
TC/HDL-C ratio	3.38 (2.78–4.24)	2.97 (2.67–3.23)	**<0.001**
Triglycerides/HDL-C ratio	2.68 (1.87–3.60)	2.17 (1.76–3.16)	0.110
LDL-C/HDL-C ratio	1.83 (1.38–2.43)	1.41 (1.16–1.76)	**<0.001**
Total bilirubin, mg/dL	1.89 (1.18–3.00)	2.79 (1.54–3.91)	**0.016**
Direct bilirubin, mg/dL	0.35 (0.27–0.49)	0.36 (0.24–0.44)	0.880
Indirect bilirubin, mg/dL	1.44 (0.88–2.61)	2.17 (1.21–3.50)	**0.010**
LDH, U/L	879.00 (618.00–1269.00)	855.00 (673.00–1153.00)	0.873
ALT, U/L	14.00 (10.25–19.00)	15.00 (10.00–19.00)	0.779
AST, U/L	39.00 (26.00–54.00)	35.00 (27.00–52.00)	0.512
Total protein, g/dL	8.21 (7.85–8.85)	8.44 (7.76–9.00)	0.215^∗^
Albumin, g/dL	4.79 (4.60–4.97)	4.79 (4.57–5.00)	0.868
Globulin, g/dL	3.43 (3.10–3.90)	3.50 (3.03–4.11)	0.200^∗^
Albumin/globulin ratio	1.39 (1.25–1.55)	1.30 (1.12–1.62)	0.380^∗^
Iron, mcg/dL	92.00 (74.00–120.80)	94.00 (67.00–124.00)	0.960
Ferritin, *η*g/mL	154.40 (72.70–248.00)	148.90 (89.33–410.80)	0.577
Urea nitrogen, mg/dL	18.00 (14.53–21.62)	15.00 (12.76–17.22)	**0.002** ^∗^
Creatinine, mg/dL	0.48 (0.38–0.63)	0.48 (0.36–0.57)	0.380
CRP, mg/L	2.37 (1.71–3.71)	2.75 (1.87–4.71)	0.128
AAT, mg/dL	68.20 (37.10–120.00)	73.20 (39.65–100.20)	0.873

RBC: red blood cells; MCV: mean cell volume; MCH: mean cell hemoglobin; MCHC: mean corpuscular hemoglobin concentration; RDW: red cell distribution width; LDH: lactate dehydrogenase; WBC: white blood cells; TC: total cholesterol; HDL-C: high-density lipoprotein cholesterol; LDL-C: low-density lipoprotein cholesterol; VLDL-C: very-low-density lipoprotein cholesterol; AST: aspartate aminotransferase; ALT: alanine aminotransferase; CRP: C reactive protein; AAT: alpha 1-antitrypsin; IQR: interquartile range. Bold values indicate significance at *P* < 0.05. All *P* values obtained by the Mann–Whitney *U* test, except for those with asterisk (^∗^), for which the independent *t*-test was used.

## Data Availability

All relevant data used to support the findings of this study are included within the article and the supplementary information file.

## Supplementary Materials

Supplementary Materials Supplementary Table 1 in the Supplementary Material for comprehensive data analysis.
