# Supplementary material for: Transforming Growth Factor Beta Receptor 3 Haplotypes in Sickle Cell Disease Are Associated with Lipid Profile and Clinical Manifestations
Source: Mediators Inflamm. 2020 Oct 13;2020:3185015. doi: 10.1155/2020/3185015 (PMC7603616; doi:10.1155/2020/3185015)
Supplement: Supplementary Materials — Supplementary Table 1 in the Supplementary Material for comprehensive data analysis. [file 3185015.f1.docx]

**Supplementary Table 1.** Baseline characteristics of individuals with SCD.

| **Parameter** | **N** | **Mean ± SD** | **Reference value*** |
| --- | --- | --- | --- |
| *Hemoglobin pattern* |  |  |  |
| Fetal hemoglobin, % | 175 | 6.80 ± 5.98 | < 2.00 |
| S hemoglobin, % | 175 | 73.70 ± 17.20 | - |
| *Hematological markers* |  |  |  |
| RBC, 10^6^/mL | 175 | 3.20 ± 0.84 | 4.00 – 5.40 |
| Hemoglobin, g/dL | 175 | 9.41 ± 1.72 | 11.30 – 16.30 |
| Hematocrit, % | 175 | 27.98 ± 5.30 | 35.00 – 49.00 |
| MCV, fL | 175 | 89.00 ± 11.61 | 80.00 – 99.00 |
| MCH, ρg | 175 | 30.10 ± 4.04 | 27.00 – 32.00 |
| MCHC, g/dL | 175 | 33.81 ± 0.93 | 31.50 – 35.50 |
| RDW, % | 175 | 21.19 ± 4.07 | 11.00 – 14.00 |
| Reticulocyte Count, % | 175 | 4.47 ± 1.90 | 0.50 – 1.50 |
| WBC, /mL | 175 | 10640.52 ± 3275.07 | 3700 – 10000 |
| Neutrophils, /mL | 175 | 5304.01 ± 2363.04 | 2500 – 7500 |
| Eosinophils, /mL | 175 | 390.87 ± 276.50 | 400 – 600 |
| Lymphocytes, /mL | 175 | 3717.92 ± 1379.72 | 720 – 4800 |
| Monocytes, /mL | 175 | 976.87 ± 519.15 | 120 – 1000 |
| Platelet count, x10^3^/mL | 175 | 381.62 ± 139.14 | 150 – 450 |
| Platelet Volume Average, fL | 175 | 8.02 ± 0.89 | 6.50 – 12.00 |
| Plateletcrit, % | 175 | 0.29 ± 0.10 | 0.22 – 0.24 |
| *Biochemical markers* |  |  |  |
| TC, mg/dL | 175 | 125.95 ± 27.04 | < 200.00 |
| HDL-C, mg/dL | 175 | 37.12 ± 9.36 | > 40.00 |
| LDL-C, mg/dL | 175 | 66.38 ± 23.46 | < 130.00 |
| VLDL-C, mg/dL | 175 | 20.37 ± 6.87 | < 40.00 |
| Triglycerides, mg/dL | 175 | 102.01 ± 34.24 | < 130.00 |
| Non-HDL-C, mg/dL | 175 | 88.33 ± 25.06 | < 145.00 |
| TC/HDL-C ratio | 175 | 3.56 ± 1.13 | - |
| Triglycerides/ HDL-C ratio | 175 | 2.97 ±1.60 | - |
| LDL-C/HDL-C ratio | 175 | 1.88 ± 0.82 | - |
| Total bilirubin, mg/dL | 175 | 2.46 ± 1.56 | < 1.20 |
| Direct bilirubin, mg/dL | 175 | 0.37 ± 0.16 | < 0.40 |
| Indirect bilirubin, mg/dL | 175 | 2.09 ± 1.51 | < 0.90 |
| LDH, U/L | 175 | 967.33 ± 422.52 | < 480 |
| ALT, U/L | 175 | 16.81 ± 9.52 | Male: < 45.00  Female: < 37.00 |
| AST, U/L | 175 | 40.80 ± 18.77 | < 42.00 |
| Total protein, g/dL | 175 | 8.33 ± 0.80 | 6.00 – 8.00 |
| Albumin, g/dL | 175 | 4.80 ± 0.33 | 3.50 – 5.50 |
| Globulin, g/dL | 175 | 3.53 ± 0.68 | 2.30 – 3.50 |
| Albumin /Globulin Ratio | 175 | 1.39 ± 0.26 | 1.00 – 2.50 |
| Iron, mcg/dL | 175 | 99.38 ± 39.10 | Male: 65 – 170  Female: 50 – 170 |
| Ferritin, ƞg/ mL | 175 | 216.45 ± 187.64 | Male: 23.90 – 336.20  Female: 11.00 – 306.80 |
| Urea nitrogen, mg/dL | 175 | 17.61 ± 5.54 | 15.00 – 45.00 |
| Creatinine, mg/dL | 175 | 0.49 ± 0.16 | 0.40 – 1.30 |
| CRP, mg/L | 175 | 3.49 ± 2.42 | < 6.00 |
| AAT, mg/dL | 175 | 77.71 ± 46.16 | 80.00 – 200.00 |
| RBC: Red blood cells; MCV: Mean cell volume; MCH: Mean cell hemoglobin; MCHC: Mean corpuscular hemoglobin concentration; RDW: Red Cell Distribution Width; LDH: Lactate dehydrogenase; WBC: White blood cells; TC: total cholesterol; HDL-C: High-density lipoprotein cholesterol; LDL-C: Low-density lipoprotein cholesterol; VLDL-C: Very low-density lipoprotein cholesterol; AST: Aspartate aminotransferase; ALT: Alanine aminotransferase; CRP: C reactive protein; AAT: Alpha 1-antitrypsin; SD: standard deviation. *References values for adolescents. | | | |
